# Mentalization Impairment Is Associated with Problematic Alcohol Use in a Sample of Young Adults: A Cross-Sectional Study

**DOI:** 10.3390/ijerph17228664

**Published:** 2020-11-22

**Authors:** Claudio Imperatori, Ornella Corazza, Angelo Panno, Raffaella Rinaldi, Massimo Pasquini, Benedetto Farina, Massimo Biondi, Francesco Saverio Bersani

**Affiliations:** 1 Cognitive and Clinical Psychology Laboratory, Department of Human Science, European University of Rome, 00163 Rome, Italy; claudio.imperatori@unier.it (C.I.); angelo.panno@unier.it (A.P.); benedetto.farina@unier.it (B.F.); 2 Department of Clinical, Pharmaceutical and Biological Sciences, University of Hertfordshire, Hatfield AL109AB, UK; o.corazza@herts.ac.uk; 3 Department of Medico-Surgical Sciences and Biotechnologies, Sapienza University of Rome, 04100 Latina, Italy; 4 Department of Anatomical, Histological, Forensic and Orthopaedic Sciences, Sapienza University of Rome, 00185 Rome, Italy; raffa.rinaldi@uniroma1.it; 5 Department of Human Neurosciences, Sapienza University of Rome, 00185 Rome, Italy; massimo.pasquini@uniroma1.it (M.P.); massimo.biondi@uniroma1.it (M.B.)

**Keywords:** problematic alcohol use, mentalization, addiction, psychopathology

## Abstract

*Background*: Alcohol is one of the most widely used drugs among adolescents and young people, and problematic alcohol use (PAU) is related to significant long-term biological, clinical, and psychosocial sequelae. Although preliminary reports have linked deficits in mentalization to increased vulnerability to addiction, no studies have specifically explored this phenomenon in relation to PAU. *Methods:* The association between mentalization impairment and PAU severity was investigated in a sample of 271 young adults (183 females, 65.9%; mean age: 23.20 ± 3.55 years; range: 18–34). Self-report measures investigating PAU and mentalization were administered to all participants. *Results:* Individuals with PAU reported a more frequent use of tobacco and illicit drugs in the last 12 months. PAU severity was negatively associated with mentalization capacity (*rho* = −0.21; *p* < 0.001), and also, when possible, confounding variables (i.e., gender, age, occupation, education, tobacco and illegal drugs use) were controlled for (*rho* = −0.17; *p* = 0.004). *Conclusion*: The present data showed that mentalization impairment is significantly associated with PAU among young adults, suggesting that it may have a role in the development and/or maintenance of alcohol use.

## 1. Introduction

Alcohol is one of the most widely used drugs among adolescents and young people [1,2]. Prolonged alcohol use can lead to forms of addiction characterized, among other things, by tolerance, craving, and abstinence [3,4]. The risk of developing alcohol dependence is increased among users who started using alcohol in adolescence [5], suggesting the early years to be a particularly sensitive period in the determination of later alcohol-related health outcomes. The fifth edition of the Diagnostic and Statistical Manual of Mental Disorders (DSM-5) summarizes the conditions of alcohol addiction into the diagnosis of Alcohol Use Disorder (AUD), which implies “a problematic pattern of alcohol use leading to clinically significant impairment or distress [...] occurring within a 12-month period” [3]. The conditions of alcohol dependence, AUD, and alcohol abuse are often referred to as forms of problematic alcohol use (PAU) [6].

PAU is increasingly recognized as a public health problem, with evidence suggesting that it can produce significant long-term biological, clinical, and psychosocial sequelae [2,6]. Several mental health problems associated with PAU have been identified, including increased rates of mood, anxiety and neurocognitive disorders [2,7,8]; PAU has also been associated with a higher incidence of various somatic pathologies (e.g., cancers, liver diseases, cardiovascular diseases) as well as with higher rates of suicide, motor vehicle accidents and aggression [2]. Considering such evidence, it is relevant to detect the underlying motivations and the psychological dimensions implicated in PAU among young individuals.

Emotional dysregulation has been suggested to represent a relevant phenomenon related to addiction, as drugs can be used as self-medications to decrease negative feelings, and dysregulate emotional states and negative affect [9,10]. Emotion regulation has been proposed to be closely related to mentalization, which has been defined as “the capacity to understand one’s own and others’ internal mental processes, such as thoughts, feelings, needs, desires, and motivations, and their relationship to behavior” [11], and it can be conceptualized as an evolutionarily prewired capacity “that refers to processes involved in reflective functioning about self-other and cognition-affect based on internal and external features” [12].

Inadequate mentalization capacity has been linked to various psychiatric conditions, including addictions [12,13,14,15]; difficulty in mentalizing internal, interpersonal, social and external stimuli has been suggested to lead to emotional dysregulation, which, in turn, can represent a trigger for substance misuse [12,13,14].

Although the research on the connection between mentalization and addiction is gaining interest, no studies, to the best of our knowledge, have specifically explored this phenomenon in relation to alcohol use in youth. Thus, the aim of the present study was to investigate the association between mentalization impairment and PAU in a sample of young adults, also controlling for relevant confounding variables.

## 2. Materials and Methods

### 2.1. Participants

The sample was made of 271 Italian young adults, 65.9% females and 34.1% males (183 females and 88 males; mean age: 23.20 ± 3.55 years; range: 18–34). The recruitment was performed between November 2019 and March 2020. Inclusion criteria were the following: (i) age between 18 and 34 years, (ii) good ability to understand written Italian, and (iii) having used alcohol in the previous 12 months. The study participants were recruited through a convenient sampling approach using advertising material posted around traditional community groups. After receiving information about the aims of the study, all patients provided written consent to participate.

Our investigation was approved by the Ethics committee of the Department of Human Neurosciences of Sapienza University of Rome (prot. 03/2019) in accordance with the Helsinki declaration’s criteria. All subjects voluntarily (i.e., they did not receive payment or other compensation) and anonymously participated in the present research. We performed a priori power analysis through G*Power 3.1 software [16], indicating that, given a probability level of 0.05, a sample size of 191 was required to provide a statistical power of 0.80 to identify a potential moderate [17] effect size (*r* = 0.20) in a two-sided test.

### 2.2. Measures

All participants were administered the Cut–Annoyed–Guilty–Eye (CAGE) questionnaire [18] and the mentalization questionnaire (MZQ) [19]. They were also asked to complete a dichotomous items (Yes/No) checklist investigating socio-demographic and clinical variables (i.e., sex, age, job, educational attainment, tobacco and illicit drugs use in the last twelve months).

The MZQ [19] is a 15-item self-administered questionnaire assessing the capability to represent and understand inner mental states in oneself and others according to the mentalization theory [20]. Respondents are asked to rate each item (e.g., “Most of the time I don’t feel like talking about my thoughts and feelings with others”) on a 5-point Likert scale (from “I disagree” to “I agree”) [19]. Lower scores on the MZQ reflect a greater mentalization deficit. In the original validation study [19], satisfactory psychometric properties (e.g., adequate internal consistency) and a four-factor solution were reported: (i) refusing self-reflection (i.e., avoidance of thinking about inner states), (ii) emotional awareness (i.e., the lack of perceiving and differentiating one’s own inner states), (iii) psychic equivalence mode (i.e., the tendency to equate inner mental states and outer reality so that everything appears to be real), and (iv) regulation of affect (i.e., the inability to modulate emotions). Furthermore, the MZQ total scores were found [21] to be positively related with electro-cortical connectivity in the default mode network (i.e., a brain network associated with mentalization capability). The Cronbach’s alpha in the present sample was 0.85 for the total score.

The CAGE questionnaire [18] is a screening tool based on 4 dichotomous (0 = no; 1 = yes) items (e.g., “Have you ever had a drink first thing in the morning to steady your nerves or to get rid of a hangover?”) widely used for the assessment of PAU [22]. The total score can range from 0 to 4, with higher values reflecting more severe alcohol problems. Although a cut-off of ≥ 2 is commonly used in the literature, a cutoff of ≥ 1 has also been commonly used in several reports [22], including in Italian samples [23,24], and it is often recommended [23,25,26]. Therefore, according to Agabio et al., [24] the present study data were evaluated using both cut-off scores. In the current sample, the Cronbach’s alpha was 0.60.

### 2.3. Statistical Analyses

Statistical analysis was performed using SPSS 18.0 statistical package for the social sciences (IBM, Armonk, NY, USA). Missing data (i.e., 0.9%) were replaced with the individual’s mean for the relevant total scale/subscale for two missing items or less [27]. Scales with three or more missing items (*n* = 0) would have been excluded from the analyses. Non-parametric tests were chosen because several of the considered variables were not normally distributed (i.e., Shapiro-Wilk test, *p* < 0.05).

As the primary analysis, the Spearman’s correlation coefficients (*rho*) between the MZQ and CAGE total scores were computed. Spearman’s correlation analysis was also performed controlling (i.e., standardized residuals) for possible confounding variables (i.e., gender, age, educational level, occupation, tobacco and illicit drugs use in the last six months).

For sensitivity analyses, differences between individuals with (+) and without (**−**) PAU (assessed according the CAGE cut-off values) were performed using two-way chi-squared (χ^2^) and the Mann–Whitney U test for dichotomous and dimensional measures, respectively. Analysis-appropriate effect sizes (Cohen’s *d* and Cramer’s *V*, respectively, for U and χ^2^ tests) were computed and converted to *r* values [28]. Effect size interpretation as small (*r* = 0.10), medium (*r* = 0.20) or large (*r* = 0.30) was performed according to Gignac and Szodorai [17].

## 3. Results

**Figure 1:**
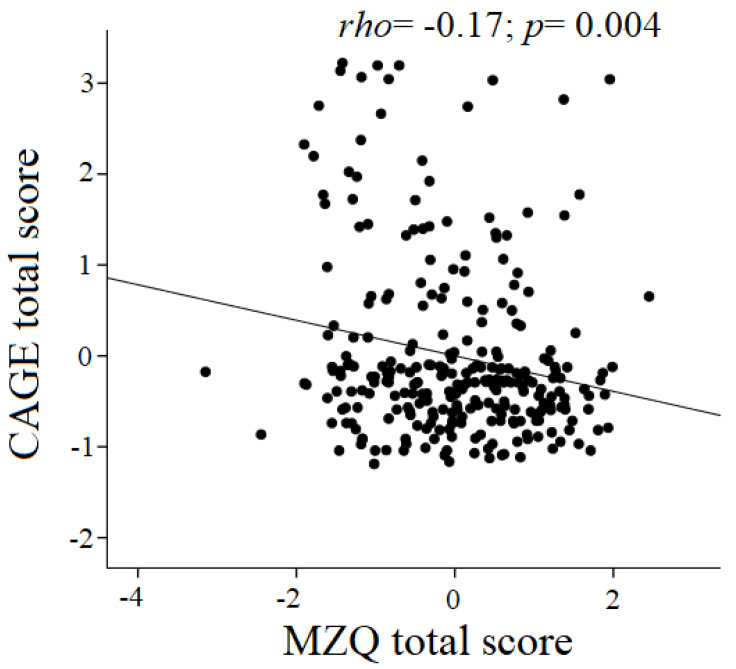
Scatterplot showing the association between CAGE and MZQ total scores after covering for confounding variables (gender, age, occupation, education, tobacco and illegal drugs use).

The clinical and socio-demographic characteristics are listed in Table 1. Among the participants, 72 (26.6%) met the criteria for PAU according to the CAGE cut-off of ≥ 1, and 37 (13.7%) according to the CAGE cut-off ≥ 2 [22]. Across all subjects, PAU severity was negatively associated with mentalization capacity (*rho* = −0.21; *p* < 0.001). Such correlation was significant also when confounding variables (i.e., gender, age, educational level, occupation, tobacco and illicit drugs use in the last six months) were controlled for (*rho* = −0.17; *p* = 0.004; Figure 1).

The differences between individuals with and without PAU according to CAGE cut-off ≥ 1 are reported in Table 2. No significant differences were observed for socio-demographic data. PAU+ participants reported more frequent tobacco use (68.1% vs. 40.7%; χ^2^ = 15.85 *p* < 0.001; *r* = 0.242) as well as more illicit drug use in the last 12 months (58.3% vs. 32.2%; χ^2^ =15.21 *p* < 0.001; *r* = 0.237). Furthermore, compared to PAU− participants, individuals with PAU showed significantly lower MZQ total scores (3.15 ± 0.76 vs. 3.51 ± 0.76; U = 5220 *p* = 0.001; *r* = 0.207) and were lower in all MZQ subscales, with the exception of the psychic equivalence dimension. Such differences in MZQ total score remained statistically significant also when the above-mentioned confounding variables were controlled for (−0.27 ± 0.99 vs. 0.10 ± 0.97; U = 5499.5 *p* = 0.003; *r* = 0.178).

Similar patterns of results were observed using CAGE cut-off ≥ 2 (Table 3), although, in this case, a gender difference was observed, with more men in the PAU+ group than in the PAU− group (48.6% vs. 29.9%; χ^2^ = 5.11 *p* = 0.024; *r* = 0.137).

As previous reports showed differences between younger (18–24) and older (25–34) young adults in the prevalence of substance misuse (e.g., [29]), age-related comparisons were performed, showing that younger young adults had increased illegal drugs use (*p* = 0.034) and decreased MZQ total score (*p* = 0.009) compared to older young adults (Supplementary Materials Table S1).

## 4. Discussion

To the best of our knowledge, this is the first study aimed at testing the relationship between mentalization capacity and PAU in a sample of young adults (mean age: 23.20 ± 3.55). The results of the present cross-sectional investigation showed that PAU severity was significantly and negatively associated with mentalization capacity, even when confounders were controlled for. Among the study participants, 26.6% and 13.7% met the criteria for PAU using CAGE ≥ 1 and CAGE ≥ 2 cut-offs, respectively, with PAU+ individuals showing significantly lower mentalization capacity than controls. Our findings are consistent with a recent study on adults (age range: 18–81) showing a significant inverse correlation of mentalization skills with food addiction and lifetime alcohol-related problems [14], as well as with epidemiological data suggesting that the prevalence of PAU among the young ranges between 2.5% and 87.9% [30]. These data are also consistent with preliminary evidence linking low mentalization capacity to addiction-related disturbances in areas different from alcohol [12,13,14,15]; it has been proposed that the use of substances in subjects with a low mentalization capacity can be conceptualized as an attempt to regulate, through specific goal-directed and rewarding behaviors, unmentalized self-states [12].

Adolescence and early adulthood are particularly sensitive periods in the determination of the later development of PAU, as well as of alcohol-related biological and clinical sequelae [5], and studies have suggested that alcohol is a potential gateway drug preceding the use and the development of addiction to other substances [31]. Subsequently, elucidating the psychological underpinning associated with PAU in such periods of life can be relevant in order to develop effective preventive interventions.

The observed relationship between decreased mentalization and increased severity of PAU may be bidirectional: on the one hand, as mentioned above, decreased mentalization may lead to emotional dysregulation which, in turn, may trigger the use of psychoactive substances; on the other hand, alcohol use may lead to a range of psychological states which can contribute to decreased mentalization. Therefore, our data raise the possibility that reduced mentalization has a role in the development and/or maintenance of alcohol use.

Of relevance, mentalization-based treatments (MBTs), aiming at increasing mentalization capacity through psychotherapy approaches, have shown effectiveness across a wide range of clinical presentations, including personality, eating and depressive disorders [11,12,32]. According to our findings, it is possible that such forms of intervention, or interventions focused on psychological constructs closely related to mentalization (e.g., empathy, metacognition, theory of mind), may play a role in the treatment of young adults with PAU. Such hypothesis has recently been preliminarily studied in subjects with concurrent borderline personality disorders and substance use disorders [33], and in mothers with histories of substance-related addiction [34,35].

The present data also highlighted that, among the participants, (i) in the previous 12 months 35.1% used cannabis, 4.8% used cocaine, and 1.1% used heroin, and (ii) the use of illicit drugs was significantly higher in subjects with PAU than in controls. Such observations add to the accumulating evidence suggesting that PAU does not occur as an isolated phenomenon, but rather it can occur in the context of complex polydrug use [2,36].

The present preliminary study has several limitations, including the following: (i) this is a cross-sectional study, thus causal relationships between the associated variables, if any, cannot be established; (ii) self-reports have been used, whose results may be affected by several biases [37]; (iii) we did not collect data on the psychiatric history of participants, although the research was specifically directed towards a non-clinical sample; (iv) a selection bias of the sample may have occurred, i.e., questionnaires might have been more accessible to certain groups of individuals (e.g., students compared to employed or unemployed people, females compared to males). Further, we assessed PAU with the extensively used CAGE scale, but we did not assess other dimensions related to PAU, such as binge drinking, withdrawal symptoms, impulsivity, or cognitive disturbances. Relatedly, in the present sample a rather low internal consistency reliability coefficient (i.e., 0.60) of CAGE was detected, and a review on 22 reports [38] showed that CAGE’s Cronbach’s alpha ranged from 0.52 to 0.90, indicating the considerable variability of this self-report. Our study therefore needs to be considered as preliminary, and future reports on the topic are encouraged to elucidate the relationship between mentalization and alcohol through a wider range of measures.

Among the strengths of the study, we include the following: (i) this is, to the best of our knowledge, the first study specifically aimed at investigating the relationship between mentalization capacity and PAU, (ii) the sample size (*n* = 271) was adequate, as indicated by the a priori power analysis performed with G*Power 3.1 software [17], and (iii) we used extensively validated assessment instruments.

## 5. Conclusions

In conclusion, the data from our study suggest that mentalization is significantly associated with PAU among young adults, and support the preliminary existing evidence highlighting the important role of reduced mentalization capacity in addiction-related phenomena. Such findings need to be further confirmed in future studies, and can be taken into consideration in terms of treatment and prevention strategies.

## Figures and Tables

**Table 1:** Descriptive statistics for the sample (*n* = 271).

Variables	
Age—M ± SD	23.20 ± 3.55
Females—*n* (%)	183 (65.9)
Occupation	
Employed—*n* (%)	57 (21.0)
Unemployed—*n* (%)	14 (5.2)
Students—*n* (%)	200 (73.8)
School attainment > 13 years—*n* (%)	124 (45.8)
Tobacco use in the last 12 months—*n* (%)	130 (48.0)
Illegal drugs use in the last 12 months—*n* (%)	106 (39.1)
Cannabis use in the last 12 months—*n* (%)	95 (35.1)
Cocaine use in the last 12 months—*n* (%)	13 (4.8)
Heroin use in the last 12 months—*n* (%)	3 (1.1)
Other illegal drugs in the last 12 months—*n* (%)	6 (2.2)
Illegal drugs polyabuse in the last 12 months—*n* (%)	10 (3.7)
CAGE ≥ 1—*n* (%)	72 (26.6)
CAGE ≥ 2—*n* (%)	37 (13.7)
CAGE total score—M ± SD	0.45 ± 0.84
MZQ—M ± SD	3.41 ± 0.77
Self-reflection—M ± SD	3.55 ± 0.89
Emotional awareness—M ± SD	3.42 ± 1.01
Psychic equivalence—M ± SD	3.27 ± 0.93
Affect Regulation—M ± SD	3.78 ± 1.07

Abbreviation: CAGE = Cut–Annoyed–Guilty–Eye questionnaire; MZQ = mentalization questionnnaire.

**Table 2:** Differences between individuals with and with and without PAU according CAGE cut-off ≥ 1.

Variables	PAU+ *n =* 72	PAU− *n =* 199	Test	*p=*	Effect Size	*r=*
Age—M ± SD	22.56 ± 2.82	23.42 ± 3.76	U = 6418.5	0.189	*d* = 0.159	0.079
Females—*n* (%)	43 (59.7%)	140 (70.4%)	χ^2^ = 2.72	0.099	*V* = 0.100	0.100
School attainment > 13 years—*n* (%)	30 (41.7%)	94 (47.2%)	χ^2^ = 0.66	0.416	*V* = 0.049	0.049
Occupation						
Employed—*n* (%)	12 (16.7%)	45 (22.6%)	χ^2^ = 2.58	0.276		
Students—*n* (%)	58 (80.6%)	142 (71.4%)			*V* = 0.098	0.138
Unemployed—*n* (%)	2 (2.8%)	12 (6.0%)				
Tobacco use (last 12 months)—*n* (%)	49 (68.1%)	81 (40.7%)	χ^2^ = 15.85	**<0.001**	***V* = 0.242**	**0.242**
Illegal drugs use (last 12 months)—*n* (%)	42 (58.3%)	64 (32.2%)	χ^2^ = 15.21	**<0.001**	***V* = 0.237**	**0.237**
MZQ Total score—M ± SD	3.15 ± 0.76	3.51 ± 0.76	U = 5220	**0.001**	***d* = 0.424**	**0.207**
Self-reflection—M ± SD	3.31 ± 0.85	3.64 ± 0.90	U = 5575.5	**0.005**	***d* =0.344**	**0.170**
Emotional awareness—M ± SD	3.04 ± 1.10	3.56 ± 0.98	U = 5087	**<0.001**	***d* =0.454**	**0.221**
Psychic equivalence—M ± SD	3.16 ± 0.92	3.31 ± 0.94	U = 6441.5	0.203	*d* = 0.154	0.077
Affect Regulation—M ± SD	3.04 ± 0.97	3.50 ± 1.07	U = 5301	**0.001**	***d* = 0.405**	**0.198**
MZQ standardized residual *—M ± SD	−0.27 ± 0.99	0.10 ± 0.97	U = 5499.5	**0.003**	***d* = 0.361**	**0.178**

* = scores after covering for confounding variables (gender, age, occupation, education, tobacco and illegal drugs use). Abbreviations: PAU = problematic alcohol use; CAGE = Cut-Annoyed-Guilty-Eye questionnaire; MZQ = mentalization questionnaire. In bold significant differences.

**Table 3:** Differences between individuals with and without PAU according CAGE cut-off ≥ 2.

Variables	PAU+ *n* = 37	PAU– *n* = 234	Test	*p*=	Effect Size	*r*=
Age—M ± SD	22.49 ± 2.55	23.31 ± 3.67	U = 3901	0.332	*d* = 0.118	0.059
Females—*n* (%)	19 (51.4%)	164 (70.1%)	χ^2^ = 5.11	0.024	*V* = 0.137	0.137
School attainment > 13 years—*n* (%)	13 (35.1%)	111 (47.4%)	χ^2^ = 1.95	0.163	*V* = 0.085	0.085
Occupation						
Employed—*n* (%)	6 (16.2%)	51 (21.8%)	χ^2^ = 1.28	0.526		
Students—*n* (%)	30 (81.1%)	170 (72.6%)			*V* = 0.069	0.097
Unemployed—*n* (%)	1 (2.7%)	13 (5.6%)				
Tobacco use (last 12 months)—*n* (%)	27 (73.0%)	103 (44.0%)	χ^2^ = 10.73	**<0.001**	***V* = 0.199**	**0.199**
Illegal drugs use (last 12 months)—*n* (%)	24 (64.9%)	82 (35.0%)	χ^2^ = 11.93	**<0.001**	***V* = 0.210**	**0.210**
MZQ Total score—M ± SD	3.01 ± 0.75	3.47 ± 0.76	U = 2793	**0.001**	***d* = 0.431**	**0.211**
Self-reflection—M ± SD	3.23 ± 0.88	3.60 ± 0.89	U = 3222	**0.012**	***d* = 0.307**	**0.151**
Emotional awareness—M ± SD	2.84 ± 1.02	3.51 ± 0.98	U = 2704	**<0.001**	***d* = 0.457**	**0.223**
Psychic equivalence—M ± SD	3.14 ± 0.85	3.30 ± 0.95	U = 3828	0.257	*d* = 0.138	0.069
Affect Regulation—M ± SD	2.77 ± 0.91	3.48 ± 1.06	U = 2636	**<0.001**	***d* = 0.477**	**0.232**
MZQ standardized residual *—M ± SD	−0.45 ± 1.04	0.07 ± 0.96	U = 2948	**0.002**	***d* = 0.386**	**0.190**

* = scores after covering for confounding variables (gender, age, occupation, education, tobacco and illegal drugs use). Abbreviations: PAU = problematic alcohol use; CAGE = Cut–Annoyed–Guilty–Eye questionnaire; MZQ = mentalization questionnaire. In bold significant differences.

## Supplementary Materials

The following are available online at https://www.mdpi.com/1660-4601/17/22/8664/s1, Table S1: Differences between younger (18–24) and older (25–34) young adults.
