# Supplementary material for: Mentalization Impairment Is Associated with Problematic Alcohol Use in a Sample of Young Adults: A Cross-Sectional Study
Source: Int J Environ Res Public Health. 2020 Nov 22;17(22):8664. doi: 10.3390/ijerph17228664 (PMC7700465; doi:10.3390/ijerph17228664)
Supplement: Supplementary file 1 [file ijerph-17-08664-s001.zip › ijerph-964602-suppl.docx]

Supplementary Materials: Mentalization Impairment Is Associated with Problematic Alcohol Use in a Sample of Young Adults: A Cross-Sectional Study

**Table S1.** Differences between younger (18–24) and older (25–34) young adults.

| **Variables** | **Younger**  ***N = 187*** | **Older**  ***N = 84*** | **Test** | ***p=*** | ***Effect Size*** | ***r=*** |
| --- | --- | --- | --- | --- | --- | --- |
| Age—M ± SD | 21.33 ± 2.04 | 27.37 ± 2.48 | U= 0.000 | **< 0.001** | *d*= 2.663 | 0.800 |
| Females—N (%) | 121 (64.7%) | 62 (73.8%) | χ^2^= 2.19 | 0.139 | *V*= 0.090 | 0.090 |
| Tobacco use (last 12 months)—N (%) | 91 (48.7%) | 39 (46.4%) | χ^2^= 0.12 | 0.733 | *V*= 0.021 | 0.021 |
| Illegal drugs use (last 12 months)—N (%) | 81 (43.3%) | 25 (29.8%) | χ^2^= 4.47 | **0.034** | ***V*= 0.128** | **0.128** |
| CAGE ≥ 1—N (%) | 55 (29.4%) | 17 (20.2%) | χ^2^= 2.50 | 0.114 | *V*= 0.096 | 0.096 |
| CAGE ≥ 2—N (%) | 30 (16.0%) | 7 (8.3%) | χ^2^= 2.92 | 0.087 | *V*= 0.104 | 0.104 |
| CAGE Total score—M ± SD | 0.50 ± 0.88 | 0.32 ± 0.73 | U= 7084.5 | 0.096 | *d*= 0.157 | 0.078 |
| MZQ Total score—M ± SD | 3.33 ± 0.77 | 3.58 ± 0.75 | U= 6288.0 | **0.009** | ***d*= 0.323** | **0.159** |
| Self-reflection—M ± SD | 3.48 ± 0.90 | 3.71 ± 0.89 | U= 6746.5 | 0.063 | *d*= 0.227 | 0.113 |
| Emotional awareness—M ± SD | 3.34 ± 0.98 | 3.61 ± 1.06 | U= 6557.0 | **0.029** | ***d*= 0.266** | **0.132** |
| Psychic equivalence—M ± SD | 3.24 ± 0.94 | 3.34 ± 0.93 | U= 7367.0 | 0.413 | *d*= 0.099 | 0.049 |
| Affect Regulation—M ± SD | 3.23 ± 1.07 | 3.71 ± 0.99 | U= 5792.5 | **<0.001** | *d*= 0.429 | **0.210** |
| Abbreviations: CAGE= Cut-Annoyed-Guilty-Eye questionnaire; MZQ= Mentalization Questionnaire. | | | | | | |
