# Supplementary material for: Mentalization Impairment Is Associated with Problematic Alcohol Use in a Sample of Young Adults: A Cross-Sectional Study
Source: Int J Environ Res Public Health. 2020 Nov 22;17(22):8664. doi: 10.3390/ijerph17228664 (PMC7700465; doi:10.3390/ijerph17228664)
Supplement: Supplementary file 1 [file ijerph-17-08664-s001.zip › ijerph-964602-suppl.pdf]

International Journal of
Environmental Research and Public Health

MDPI

Supplementary Materials: Mentalization Impairment Is Associated with Problematic Alcohol Use in a Sample of Young Adults: A Cross-Sectional Study

Table S1. Differences between younger (18–24) and older (25–34) young adults.

**Variables**	**Younger** *N = 187*	**Older** *N = 84*	**Test**	*p=*	*Effect Size*	*r=*
Age—M ± SD	21.33 ± 2.04	27.37 ± 2.48	U= 0.000	**< 0.001**	*d*= 2.663	0.800
Females—N (%)	121 (64.7%)	62 (73.8%)	χ^2= 2.19	0.139	*V*= 0.090	0.090
Tobacco use (last 12 months)—N (%)	91 (48.7%)	39 (46.4%)	χ^2= 0.12	0.733	*V*= 0.021	0.021
Illegal drugs use (last 12 months)—N (%)	81 (43.3%)	25 (29.8%)	χ^2= 4.47	**0.034**	***V*= 0.128**	**0.128**
CAGE ≥ 1—N (%)	55 (29.4%)	17 (20.2%)	χ^2= 2.50	0.114	*V*= 0.096	0.096
CAGE ≥ 2—N (%)	30 (16.0%)	7 (8.3%)	χ^2= 2.92	0.087	*V*= 0.104	0.104
CAGE Total score—M ± SD	0.50 ± 0.88	0.32 ± 0.73	U= 7084.5	0.096	*d*= 0.157	0.078
MZQ Total score—M ± SD	3.33 ± 0.77	3.58 ± 0.75	U= 6288.0	**0.009**	***d*= 0.323**	**0.159**
Self-reflection—M ± SD	3.48 ± 0.90	3.71 ± 0.89	U= 6746.5	0.063	*d*= 0.227	0.113
Emotional awareness—M ± SD	3.34 ± 0.98	3.61 ± 1.06	U= 6557.0	**0.029**	***d*= 0.266**	**0.132**
Psychic equivalence—M ± SD	3.24 ± 0.94	3.34 ± 0.93	U= 7367.0	0.413	*d*= 0.099	0.049
Affect Regulation—M ± SD	3.23 ± 1.07	3.71 ± 0.99	U= 5792.5	**<0.001**	*d*= 0.429	**0.210**

Abbreviations: CAGE= Cut-Annoyed-Guilty-Eye questionnaire; MZQ= Mentalization Questionnaire.
